# Multifunction Applications of Filtering Dielectric Resonator Antenna Based on Liquid Crystal

**DOI:** 10.3390/s24010115

**Published:** 2023-12-25

**Authors:** Ke Xia, Lei Zhang, Haifeng Zhang

**Affiliations:** College of Electronic and Optical Engineering & College of Flexible Electronics (Future Technology), Nanjing University of Posts and Telecommunications, Nanjing 210023, China1221025101@njupt.edu.cn (L.Z.)

**Keywords:** temperature sensor, liquid crystal, hairpin bandpass filter, dielectric resonator antenna

## Abstract

In this paper, a new type of multifunctional device is realized by designing a filtering dielectric resonator antenna (FDRA) with liquid crystal (LC). The LC is encapsulated by glass plates and placed between the feeding network and the ground. Firstly, the resonance frequencies of the hairpin bandpass filter (|*S*_11_| is less than −10 dB) move simultaneously when the dielectric constant of LC changes at different temperatures. Then, the hairpin bandpass filter is extended to an FDRA, and the influence of the dielectric constant of LC on the antenna performance parameters is realized to the function of the temperature sensor. The results show that the dielectric constant of LC has an approximately linear relationship with the resonance frequencies of the FDRA. Simultaneously, the axial ratio, gain, antenna efficiency, E-field distribution, and pattern of the FDRA have changed significantly. Furthermore, the FDRA mainly works in the frequency range of 4.65~5.53 GHz, which has good antenna performance and filtering characteristics. Taking resonance frequency *f_x_* as an example, its sensitivity, maximum *FOM*, minimum detection limit, and minimum resolution are determined to be 95 GHz/RIU, 0.5, 0.1, and 9.68, respectively. The multifunctional device provides a novel approach and solution for the transmission of antenna signals and temperature measurements.

## 1. Introduction

As a device that converts a physical quantity or signal into another physical quantity or signal, the sensors have various types of applications, such as photoelectric sensors [1], pressure sensors [2], and acceleration sensors [3]. The primary function is to convert environmental information into digital signal output, which plays an important role in different scenarios [4]. At present, the application scenarios for sensors are expanding, showcasing a shift from traditional single-function devices towards integrated and diversified systems [5]. Among these, temperature sensors (TSs) hold significant prominence, being extensively employed in the industrial, agricultural, medical, and environmental sectors [6,7].

TSs play an important role in various fields, providing temperature data for control, monitoring, and regulation, thereby improving system efficiency, reliability, and safety. However, some TSs that employ traditional substances have drawbacks, such as thermistors utilizing copper oxide having a relatively slow response to temperature changes [8,9], thermocouple TSs composed of two different metal wires having restrictive measurement accuracy [10], and using platinum resistance as a TS having higher costs [11]. In light of material science and engineering advancements, researchers have been exploring the utilization of new materials for TS development [12,13]. An emerging and captivating research direction involves harnessing the unique characteristics of liquid crystal (LC) to enable temperature detection. LC, as a particular class of materials, demonstrates distinct ordered arrangement states in response to temperature variations, leading to changes in dielectric constants [14,15]. Accordingly, the design of a comprehensive structure around the combination of LC and other related devices can realize the function of temperature measurement.

With the rise in interdisciplinary disciplines, the new technology of sensor and antenna combinations has become a research hotspot [16,17]. The popularity of antenna sensors has increased rapidly due to their low price, ease of production, passive functions, and ability to sense various signals [18]. In addition, antenna sensors have developed into a new method for measuring a wide range of physical properties, including glucose [19], humidity [20], pH [21], fracture [22], and strain [23]. The multifunctional devices combined with sensors and antennas can leverage advanced antenna technology to support various communication standards and protocols while integrating a plethora of sensors and electronic modules to enable widespread deployment in diverse fields. For instance, Azaro et al. developed multifunctional devices based on antennas to facilitate wireless services such as localization, voice and data communications, and emergency calls [24]. Through rigorous experimentation and numerical simulations, the feasibility of using the designed multifunctional device in the automobile rescue management system is validated.

At the same time, the problem of how to combine new TSs with antenna technology has attracted wide attention in academic and engineering fields. For instance, Sanders et al. derived the relationship between resonance frequency shift and temperature variation based on the transmission line model [25]. By comparing the measured temperature–frequency relationship with the theoretical prediction, they verified that the designed microstrip patch antenna can function as a TS. Moreover, it is noteworthy that the long-term stability of the proposed antenna sensor still deserves further discussion. Tchafa et al. investigated the use of a single antenna with two fundamental resonance frequencies for simultaneous strain and temperature sensing [23]. The experimental results confirmed the theoretical prediction that the normalized resonance frequency shift is linearly proportional to the applied strain and temperature changes. However, verification is still needed in terms of using high-temperature substrate materials to improve the maximum operating temperature.

Compared with patch antennas, dielectric resonator antennas (DRAs) offer the advantages of smaller size and better stability [26,27], which makes them favorable for integration into microdevices and long-term monitoring applications. Dielectric resonators (DRs) not only exhibit different properties under different physical size conditions [28] but also can apply some special properties to extend the additional effects [29,30]. Therefore, combining DRAs with TSs is a promising research direction that deserves exploration. For instance, to measure temperatures in harsh environments, an integrated TS based on a wireless passive resonator antenna of low-temperature co-fired ceramic is designed. Tan et al. proposed a resonator–antenna integrated microstrip antenna TS based on low-temperature co-fired ceramic and explored the change of |*S*_11_| from 50 to 400 °C, showing great potential for temperature monitoring in harsh environments [31]. Although TSs using antenna technology have been widely used in practice, the high sensitivity, miniaturization, and integration of sensors are still worthy of further study, especially based on LC.

This paper presents the design of a TS achieved by utilizing the impact of varying dielectric constants of LC at different temperatures on the performance of the DRA. Firstly, there is a good linearity between the dielectric constant of LC and the resonant frequency of the hairpin bandpass filter (HBF). Then, a DRA fed by slot coupling mode is designed, and an HBF is added to the feeding network to increase the number of resonance points to improve the reliability of temperature measurement. The relationship between the relevant parameters of the designed antenna and the dielectric constant of LC is studied. The calculation results show that the movement of the three peak resonant frequencies of the filtering dielectric resonator antenna (FDRA) depends on the change of the dielectric constant of the LC and has good linearity. The curves of axial ratio (AR), gain, and antenna efficiency of FDRA have shifted, and the electric field distribution and mode have also changed significantly. As a multifunctional device, this design can be described as a high-performance FDRA that also facilitates temperature detection using LC material.

## 2. Multifunctional Device Design

### 2.1. Configuration of the HBF

**Figure 1:**
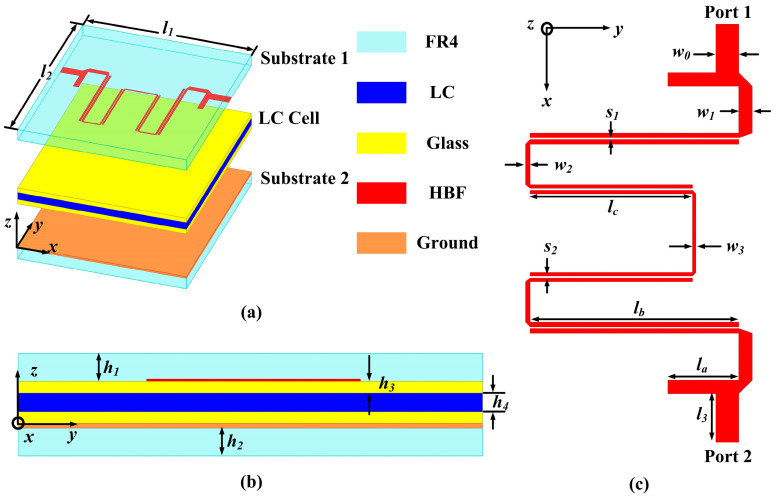
Structure diagram of the HBF based on LC: (**a**) the perspective view of the HBF, (**b**) the side view of the HBF based on LC, and (**c**) the detailed configuration and sizes of the HBF.

The proposed TS consists of substrate 1, substrate 2, HBF, glass plates, LC, and ground plane, as illustrated in Figure 1a. Substrates 1 and 2 are made of FR4 (*ε_r_* = 4.4 and tan*δ* = 0.02) [32] (a composition of glass fiber fabric and epoxy resin). The HBF is fabricated on the lower surface of substrate 1, while the metal ground is printed on the upper surface of substrate 2. The LC medium selected for this sensor consists of various substances (4′-Pentyl-4-cyano-biphenyl, 4′-Alkyl-4-isothiocyanato-phenylcyclohexanes, 4″-Alkyl-2′ and 5′fluoro-phenyl-4 alkyl-biphenyl-tolanes), with its dielectric constant (*ε*_//_ = 3.1, *ε*_⊥_ = 2.5, and tan*δ* < 0.06) capable of being adjusted within the temperature range of −20~50 °C (*ε*_//_ is the permittivity parallel and *ε*_⊥_ is perpendicular to the long axes of the LC molecules) [33]. Two glass plates encapsulate the LC, forming an LC cell, as depicted in Figure 1b. For calculation, the maximum and minimum dielectric constants of the LC (*ε*_LC_) are set as 3.1 and 2.5 [33], respectively. Moreover, the specific parameters of the HBF are provided in Figure 1c, with detailed parameter sizes for the HBF based on LC listed in Table 1.

**Figure 2:**
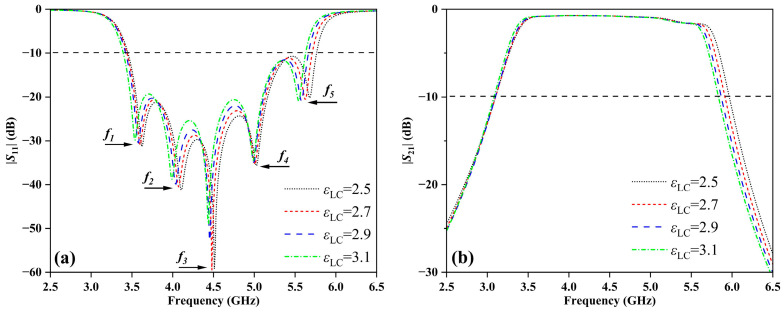
Simulation result of the *S*-parameter of the HBF with different values of *ε*_LC_: (**a**) |*S*_11_| and (**b**) |*S*_21_|.

The *S*-parameter of the HBF with different dielectric constants of LC (*ε*_LC_) is shown in Figure 2. The reflection coefficient of the input port, denoted as |*S*_11_|, quantifies the extent of the input signal’s reflection from Port 1 back to Port 1. Conversely, |*S*_21_|, the transmission parameter, reflects the degree of signal transmission from Port 1 to Port 2. Figure 2a reveals that the peak resonance frequencies (*f*_1~5_) of the HBF undergo noticeable shifts in response to variations in *ε*_LC_. Simultaneously, the impedance bandwidth of the HBF also experiences significant changes. For instance, compared with the frequency range of 3.39~5.83 GHz observed when *ε*_LC_ equals 2.5, the impedance bandwidth shifts to 3.34~5.65 GHz for a dielectric constant of 3.1. Furthermore, Figure 2b shows that the range of |*S*_21_| also changes.

The sensitivity (*S*), *Q*-factor, figure of merit (*FOM*), detection limit (*DL*), and resolution (*RS*) are important parameters for evaluating a sensor. The following formulas are usually used to measure the performance of the sensor [34]:

(1) $$S=\frac{\Delta f}{\Delta n}$$

(2) $$Q=\frac{f_{T}}{FWHM}$$

(3) $$FOM=\frac{S}{FWHM}$$

(4) $$DL=\frac{f_{T}}{20SQ}$$

(5) $$RS=\frac{FWHM}{1.5\times {\left(\frac{\Delta f}{FWHM}\right)}^{0.25}}$$

where Δ*f* refers to the frequency, and the change of refractive index (Δ*n*) is the change of dielectric constant of LC, while *f_T_* is the peak resonant frequency, and FWHM implies the full width at half maxima of the peak resonant frequency.

**Figure 3:**
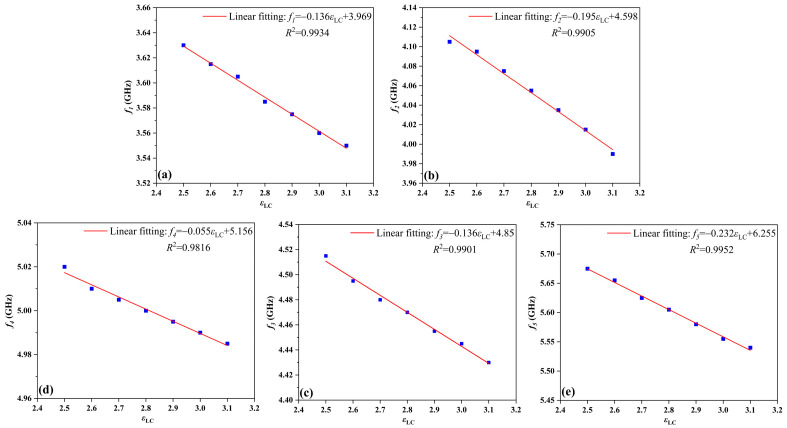
Simulated peak resonant frequencies of the HBF for various dielectric constants of the LC (*ε*_LC_): (**a**) the curve of peak resonant frequency *f*_1_, (**b**) the curve of peak resonant frequency *f*_2_, (**c**) the curve of peak resonant frequency *f*_3_, (**d**) the curve of peak resonant frequency *f*_4_, and (**e**) the curve of peak resonant frequency *f*_5._

To further investigate the impact of temperature regulation, Figure 3 displays the relationship between the peak resonant frequencies of the temperature sensing and the dielectric constant of the LC (*ε*_LC_). To mitigate interference, a threshold of |*S*_11_| = −10 dB is considered such that, during sensing operation, the values of |*S*_11_| must fall below this threshold. For instance, the data of the peak frequency *f_5_* are extracted from Figure 2a, and the linear fitting function of the peak frequency point with the dielectric constant of LC is *f_5_* = −0.232*ε*_LC_ + 6.255. As shown in Figure 3d, the goodness of fit *R*^2^ [34] is 0.9952, and the sensing sensitivity is 232 GHz/RIU, calculated by Equation (1). The high linear goodness of fit provides high implementability and high predictability for high-precision sensing applications. In other words, the peak resonant frequencies of the HBF can be adjusted by changing the dielectric constant of the LC at different temperatures. Additionally, the HBF based on LC successfully fulfills its function of temperature detection, thereby providing a solid foundation for its extension into the sensor combined with DRA.

### 2.2. Configuration with FDRA

**Figure 4:**
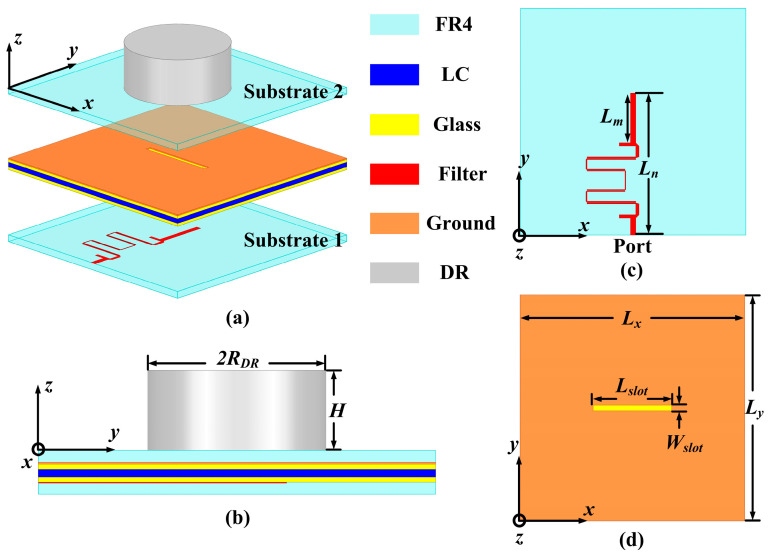
Structure diagram of the TS based on FDRA: (**a**) the perspective view of the TS, (**b**) the side view of the TS, (**c**) the top view of the feeding network with HBF, and (**d**) the top view of the ground with the slot.

Building upon the foundation of the designed HBF based on LC, the proposal is extended to introduce a TS based on FDRA, which is composed of substrate 1, substrate 2, DR, filtering feeding network, glass plates, LC, and metal ground, as illustrated in Figure 4a. As shown in Figure 4b, the cylindrical DR, composed of Al_2_O_3_ ceramic (*ε_r_* = 9.5, tan*δ* = 0.003) [35], is positioned above substrate 2. The feeding network, based on the HBF, is printed on the upper surface of substrate 1 (FR4), while the metal ground is printed on the lower surface of substrate 2 (FR4). If a probe feeding mode is used, it inevitably causes a certain destructive impact on the glass plates and LC; therefore, adopting the slot feeding method to excite the DR is a more appropriate choice. By utilizing the slot feeding mode, a more compact structural design can be achieved on the DR, which is beneficial for the integration and miniaturization of the TS. Furthermore, a slot of dimensions *W_slot_* × *L_slot_* is created in the metal ground. The detailed parameters of the feeding network and the metal ground are provided in Figure 4c,d. In addition, the relevant properties of the Al_2_O_3_ ceramic DR are summarized in Table 2, and specific parameters of the proposed TS based on FDRA are listed in Table 3.

For some regular basic shapes, there are some empirical formulas for the resonant frequency of DRA [37]. For example, for the basic HEM_11δ_ and TM_01δ_ modes of the cylindrical DRA, the resonant frequencies can be estimated by the following formulas [38]:

(6) $${\left(k_{0}R_{DR}\right)}_{{\mathrm{HEM}}_{11\delta }}=\frac{6.324}{\sqrt{\varepsilon_{r}+2}}\left(0.27+0.36\left(\frac{R_{DR}}{2H}\right)+0.02{\left(\frac{R_{DR}}{2H}\right)}^{2}\right)$$

where 0.33 ≤ *R_DR_*/*H* ≤ 5

(7) $${\left(k_{0}R_{DR}\right)}_{{\mathrm{TEM}}_{01\delta }}=\sqrt{3.83^{2}+{\left(\frac{\pi R_{DR}}{2H}\right)}^{2}}/\sqrt{\varepsilon_{r}+2}$$

where 0.4 ≤ *R_DR_*/*H* ≤ 6

(8) $$f_{{\mathrm{HEM}}_{11\delta }}=\frac{c}{2\pi R}{\left(k_{0}R_{DR}\right)}_{{\mathrm{HEM}}_{11\delta }}$$

(9) $$f_{{\mathrm{TEM}}_{01\delta }}=\frac{c}{2\pi R}{\left(k_{0}R_{DR}\right)}_{{\mathrm{TEM}}_{01\delta }}$$

where *R_DR_*, *H*, and *ε_r_* denote the radius, height, and dielectric constant of the cylindrical DR located on a metal ground, respectively, and *k_0_* denotes the wavenumber (the *k_0_* for the free-space propagation constant and *c* for the speed of light in vacuum). These formulas provide good guidance in the early design of cylindrical DR. Substituting *R_DR_* = 9 mm, *H* = 8 mm, *ε_r_* = 9.5 into the above Equations (6)~(9), the values of $f_{{\mathrm{HEM}}_{11\delta }}$ and $f_{{\mathrm{TEM}}_{01\delta }}$ are calculated to be 4.73 GHz and 6.59 GHz, respectively. However, the aforementioned analysis does not consider the influence of the feeding method on the DR. Moreover, the frequency characteristics of the cylindrical DR are affected by the slot feeding mode, inducing a certain offset that alters the operating frequency band. Consequently, the calculated values from Equations (6)~(9) can only serve as design references for the cylindrical DR.

**Figure 5:**
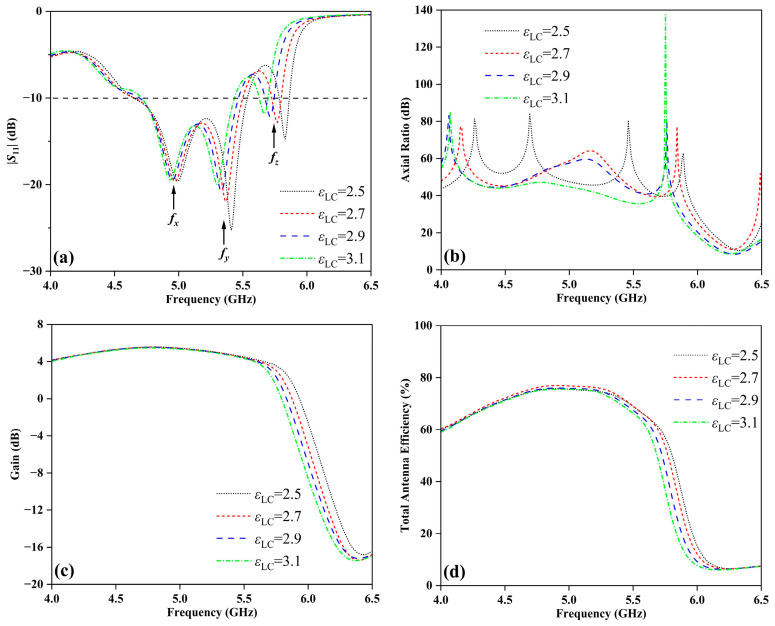
Simulated electromagnetic performance of the designed TS based on FDRA with different *ε*_LC_: (**a**) |*S*_11_|, (**b**) AR, (**c**) gain, and (**d**) total antenna efficiency.

The AR of an antenna is a quantitative measure used to describe the shape of the polarization ellipse, which is formed by the electric field vectors over one full cycle of propagation. The gain is a measure of its ability to direct or concentrate radio frequency energy in a particular direction or pattern. The total antenna efficiency is expressed as a ratio of the total power radiated by the antenna to the input power supplied to the antenna. Figure 5 presents performance parameter curves of the TS based on FDRA under different dielectric constants of LC (*ε*_LC_). As shown in Figure 5a, the TS based on FDRA has three peak resonance frequencies (*f_x_*, *f_y_*, and *f_z_*) by varying the values of *ε*_LC_. Simultaneously, the impedance bandwidths of the FDRA have also shifted significantly. Notably, Figure 5b demonstrates a pronounced variation in AR with changes in *ε*_LC_. Additionally, Figure 5c,d illustrate that the gain and total antenna efficiency remain relatively stable within the frequency range of 4~5.5 GHz while exhibiting notable deviations during 5.5~6.5 GHz.

**Figure 6:**
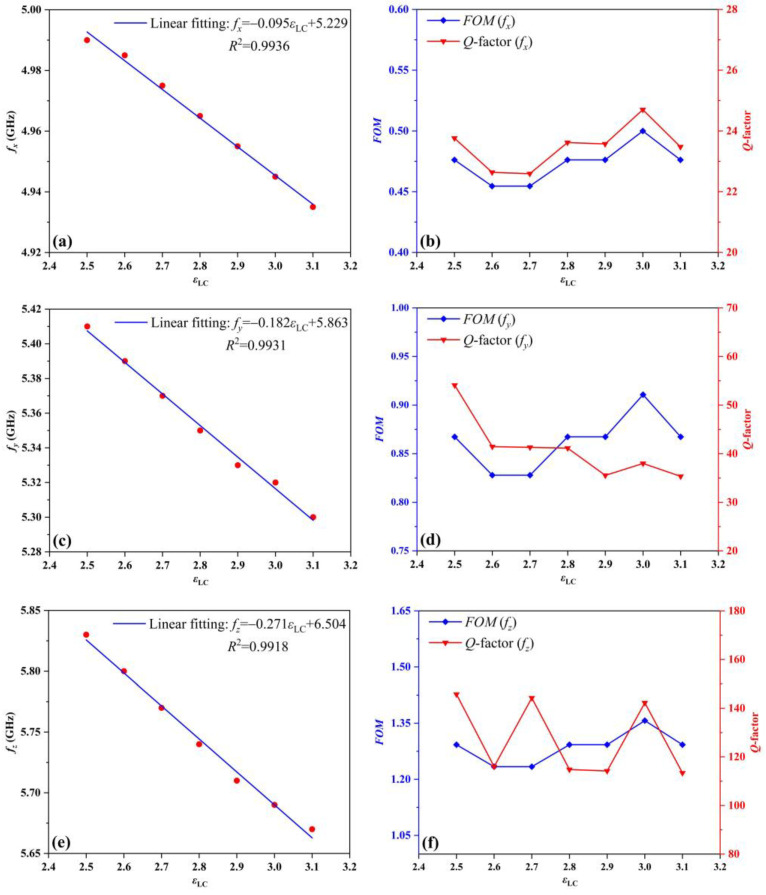
In the case of the TS based on FDRA for various dielectric constants of the LC (*ε*_LC_): (**a**) the linear fit of peak resonance frequencies (*f_x_*), (**b**) the *FOM* and *Q*-factor distribution (*f_x_*) of analytes, (**c**) the linear fit of peak resonance frequencies (*f_y_*), (**d**) the *FOM* and *Q*-factor distribution (*f_y_*) of analytes, (**e**) the linear fit of peak resonance frequencies (*f_z_*), and (**f**) the *FOM* and *Q*-factor distribution (*f_z_*) of analytes.

To further investigate the impact of temperature regulation, Figure 6 illustrates the relationship between the peak resonant frequencies of the TS and the dielectric constant of LC (*ε*_LC_). It is observed that the changes in the three peak resonance frequencies decrease as the values of *ε*_LC_ increase. Taking the peak resonance frequency *f_x_* as an example, by extracting the frequency data in Figure 5a, the linear fitting function of the peak resonance frequency *f_x_* with the change of *ε*_LC_ is calculated as *f_x_* = −0.095*ε*_LC_ + 5.229. As shown in Figure 6a, the goodness of fit *R^2^* is 0.9936, and the sensing sensitivity is 95 GHz/RIU. To evaluate the sensing performance of the antenna more comprehensively, the values of *Q*-factor and *FOM* at *ε*_LC_= 2.5, 2.6, 2.7, 2.8, 2.9, 3, and 3.1 are calculated by Equations (2) and (3). As shown in Figure 5b, the values of *FOM* are basically maintained around 0.47, and the maximum value can reach 0.5. In addition, the maximum and minimum values of *Q*-factor are 24.7 and 22.6, respectively. It is worth noting that the minimum *DL* and minimum *RS* calculated by Equations (4) and (5) are 0.1 and 9.68, respectively. Consequently, the peak resonant frequencies of FDRA can be adjusted by manipulating the dielectric constant of LC at different temperatures.

### 2.3. Results and Discussion

**Figure 7:**
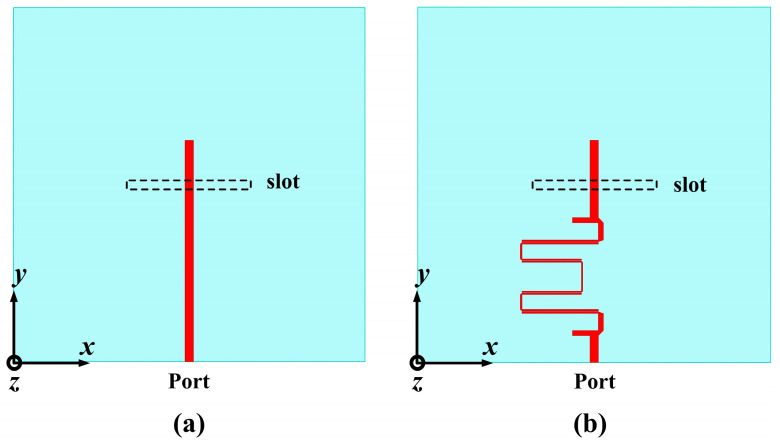
Bottom views of the DRA with or without HBF: (**a**) the feeding network without HBF and (**b**) the feeding network with HBF.

**Figure 8:**
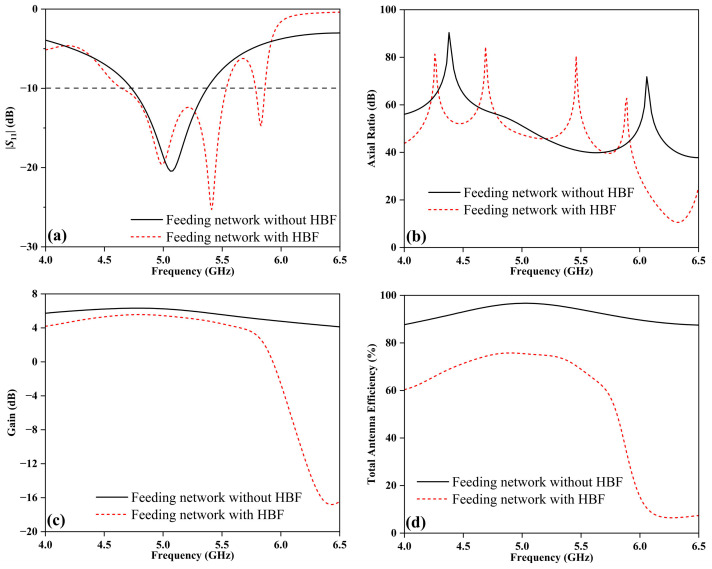
Simulated results of DRA without or with HBF when *ε*_LC_ = 2.5: (**a**) |*S*_11_|, (**b**) AR, (**c**) gain, and (**d**) total antenna efficiency.

To investigate the role of the HBF, the DRAs are studied using two different feeding methods in the feeding network, as depicted in Figure 7. When the dielectric constant of the LC (*ε*_LC_) is 2.5, the two cases of the feeding network with or without the HBF are simulated and compared, and the results are shown in Figure 8. It is evident from Figure 8a that, when *ε*_LC_ is 2.5, the impedance bandwidth of the DRA without the HBF is 12.7% (4.73~5.37 GHz), with only one peak resonance frequency. In contrast, the DRA with the HBF exhibits an impedance bandwidth of 17.5% and 1.4% (4.65~5.54 GHz and 5.78~5.86 GHz), respectively. Importantly, the addition of the HBF in the feeding network increases the number of peak resonance frequencies to three, thereby enhancing the stability of the detection process. Furthermore, Figure 8b demonstrates that the designed DRA achieves values of AR greater than 10 dB, indicating favorable linear polarization performance. Figure 8c reveals a slight decrease in gain by 0.8 dB within the range of 4~5.7 GHz after incorporating the HBF, rapidly dropping below −10 dB outside the passband. The simulated total antenna efficiencies of the two designed DRAs are presented in Figure 8d, with maximum efficiencies of 96.7% and 75.7%, respectively. Overall, the integration of an HBF into the feeding network enhances the number of peak resonance frequencies for the DRA, which is beneficial for the application of the TS.

**Figure 9:**
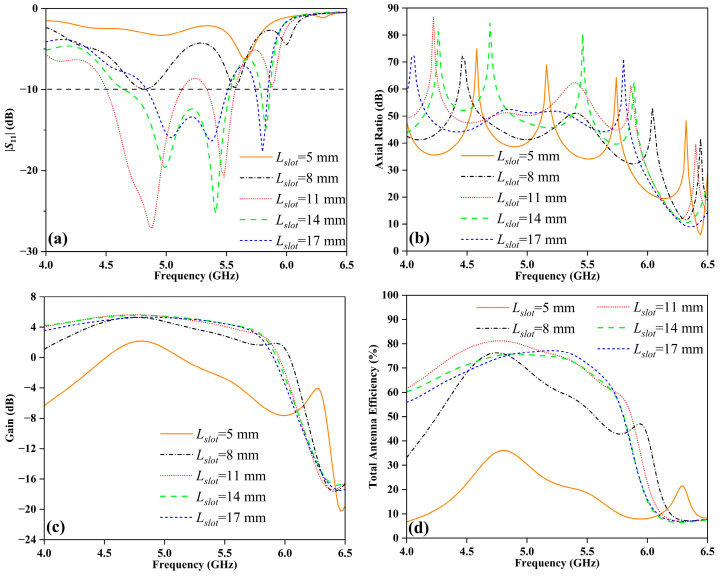
Simulated results of the TS based on FDRA for different values of *L_slot_*: (**a**) |*S*_11_|, (**b**) AR, (**c**) gain, and (**d**) total antenna efficiency.

The impact of slot size (*L_slot_*), a critical parameter, on the performance of the TS based on FDRA is thoroughly examined. Figure 9a shows that when the *L_slot_* is set to 5 mm and 8 mm, the values of |*S*_11_| remain above −10 dB within the range of 4~6.5 GHz, which hampers the detection of peak resonance frequencies. In other words, the input signal cannot be fully transmitted to the DR, resulting in an uneven field distribution in the resonator and deterioration of the impedance matching of FDRA, which is not enough to meet the application requirements. Conversely, when *L_slot_* is increased to 14 mm and 17 mm, there are three peak resonance frequencies observed, in contrast to the two peak resonance frequencies when *L_slot_* is 11 mm. This indicates that increasing the length of the gap reduces the peak resonance frequencies of the FDRA. Figure 9b illustrates that varying the length of the gap affects the AR. The increase in gap length leads to a more uniform E-field distribution, subsequently reducing the disparity between different polarization components and causing the peak values of AR to shift towards lower frequencies. Figure 9c,d demonstrate insignificant differences in gain and antenna efficiency when *L_slot_* is set to 11 mm, 14 mm, and 17 mm. However, when *L_slot_* is 5 mm, the excessively small slot directly impacts energy transmission and coupling efficiency.

**Figure 10:**
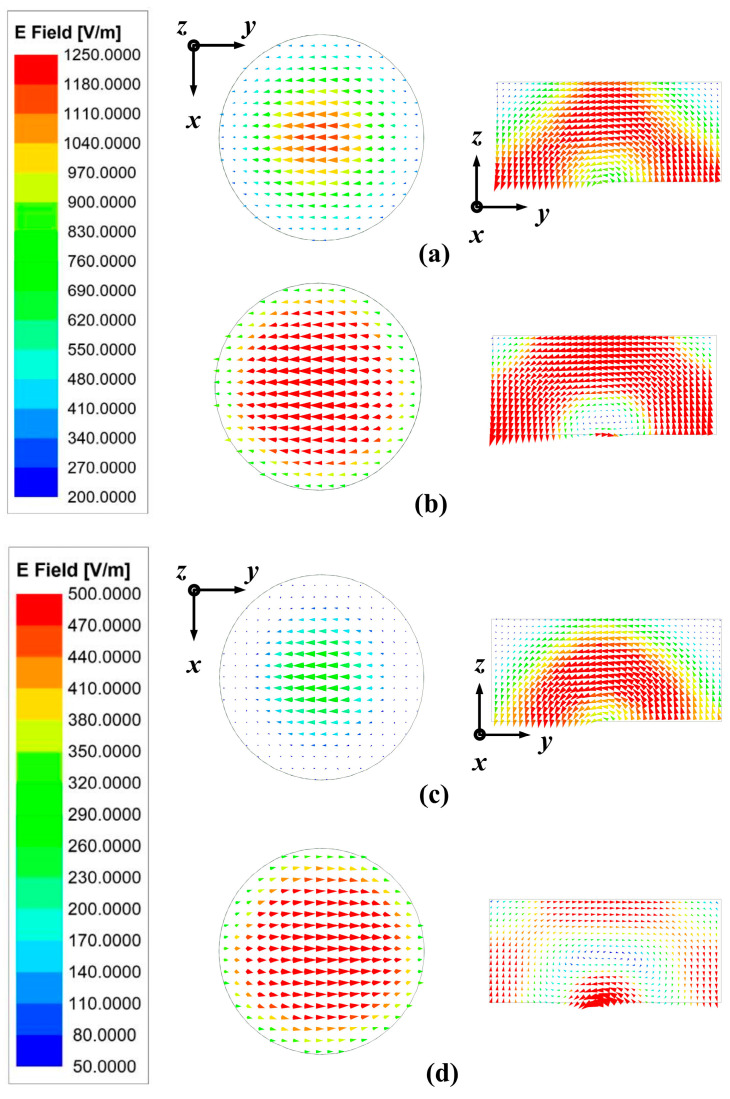
Side and top views of simulated E-field distributions of the proposed TS based on FDRA: (**a**) at 4.99 GHz when *ε*_LC_ = 2.5, (**b**) at 4.99 GHz when *ε*_LC_ = 3.1, (**c**) at 5.82 GHz when *ε*_LC_ = 2.5, and (**d**) at 5.82 GHz when *ε*_LC_ = 3.1.

Figure 10 presents the top and side views of the vector E-field distribution of the structure at frequency points of 4.99 GHz and 5.82 GHz, which confirm that the FDRA is excited in the HEM_11δ_ mode. Additionally, by assigning 2.5 and 3.1 to *ε*_LC_, respectively, the E-field distribution has changed significantly. It is intuitively proved that the change of the dielectric constant of LC under different temperatures substantially affects the E-field distribution of the FDRA.

**Figure 11:**
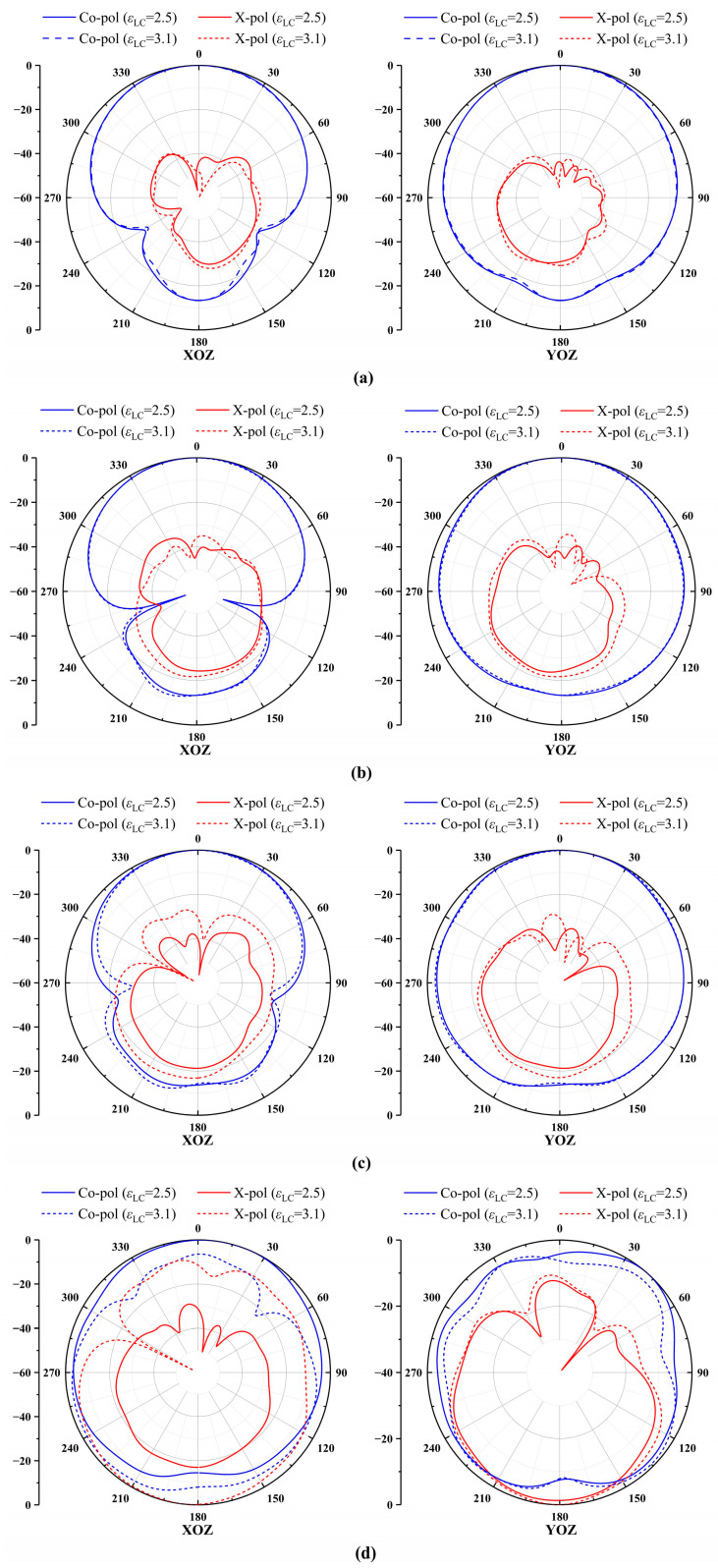
Simulated normalized patterns of the TS based on FDRA: (**a**) 4.99 GHz, (**b**) 5.41 GHz, (**c**) 5.83 GHz, and (**d**) 6.25 GHz.

To investigate the influence range of the dielectric constant of the LC (*ε*_LC_), the radiation mode of the antenna is studied and visualized in Figure 11. The normalized simulated radiation patterns at three frequency points (4.99 GHz, 5.41 GHz, and 5.83 GHz) within the passband are selected for analysis. It is evident that the values of *ε*_LC_ can alter the pattern parameters of the DRA. Figure 11d displays the simulated radiation pattern at a frequency point (6.25 GHz) outside the passband, indicating that the influence of *ε*_LC_ on the radiation of the FDRA extends beyond the passband and significantly affects it.

Table 4 provides a comprehensive comparison of various indicators of the FDRA proposed in this paper with other reported FDRAs. 

It is worth highlighting that, in comparison to antennas [40,41,42], the proposed FDRA exhibits a significantly larger impedance bandwidth. Furthermore, Table 5 offers a concise summary and comparative analysis of the TS introduced in this paper in contrast to other reported antenna TSs. The proposed FDRA represents an ongoing evolution, contributing valuable insights to the development of antenna TSs. These insights hold substantial significance for researchers and engineers engaged in related research domains.

## 3. Conclusions

In this paper, an FDRA based on LC is designed that incorporates the capability for temperature detection. Firstly, the size parameters of the HBF are reasonably designed by selecting the appropriate LC, whose dielectric constant is controlled by temperature. The results reveal that, as the dielectric constant of the LC (*ε*_LC_) increases, the peak resonant frequencies of the HBF gradually shift towards lower frequencies. To further explore the physical properties, the HBF is extended to FDRA and combined with LC to design an antenna sensor. When the proposed sensor is operational, the peak resonant frequencies exhibit high sensitivity to the values of *ε*_LC_, allowing for precise calculation of temperature variations based on changes in antenna performance. Notably, the peak resonance frequencies of both the HBF and the FDRA exhibit a strong linear relationship with variations in the values of *ε*_LC_, affirming their efficacy for temperature measurement. Taking resonance frequency *f_x_* as an example, its sensitivity, maximum *FOM*, minimum detection limit, and minimum resolution are determined to be 95 GHz/RIU, 0.5, 0.1, and 9.68, respectively. The proposed TS offers several advantages, including a simple structure, low cost, and practical applicability in temperature sensing. This work presents a novel approach to combining TSs with antennas, opening up new possibilities for their integration in various applications.

## Figures and Tables

**Table 1:** Value of the parameters.

*l*_1_	*l*_2_	*l*_3_	*l_a_*	*l_b_*	*l_c_*	*s*_1_	*s*_2_
20 mm	17.28 mm	2 mm	2.94 mm	8.66 mm	6.74 mm	0.08 mm	0.09 mm
*h*_1_	*h*_2_	*h*_3_	*h*_4_	*w*_0_	*w*_1_	*w*_2_	*w*_3_
0.8 mm	0.8 mm	0.1 mm	0.2 mm	0.95 mm	0.56 mm	0.19 mm	0.14 mm

**Table 2:** Properties of the DR (Al_2_O_3_ ceramic) [36].

*ε_r_*	9.5
Density (g/cm^3^)	3.9
Flexural strength (Mpa)	340
Compressive strength (Mpa)	3600
Dielectric strength (KV/mm)	25
Thermal conductivity (W/m·K)	27
Modulus of elasticity (Gpa)	380
Highest application temperature (°C)	1750

**Table 3:** Parameters of the proposed TS based on FDRA.

*R_DR_*	*H*	*L_m_*	*L_n_*
9 mm	8 mm	8.72 mm	25 mm
***L_x_***	***L_y_***	***L_slot_***	***W_solt_***
40 mm	40 mm	14 mm	1 mm

**Table 4:** Comparison with other FDRAs.

Refs.	*ε_r_*	Size	|*S*_11_|	Peak Gain (dBi)	Extra Filtering Circuit	Element Number	Polarization
[39]	9	0.64 × 0.64 × 0.24*λ*_c_^3^	22.07%	6.6	Yes	1	LP
[40]	10.2	0.307 × 0.266 × 0.092*λ*_c_^3^	6.13%	5.33	Yes	1	LHCP
[41]	10	0.46 × 0.46 × 0.158*λ*_c_^3^	4.5%	5.1	No	1	RHCP
[42]	10	1.93 × 1.93 × 0.14*λ*_c_^3^	6.9%	6.7	No	2 × 2	LP
Proposed	9.5	0.7 × 0.7 × 0.18*λ*_c_^3^	17.5% and 1.4%	5.57	Yes	1	LP

*λ*_c_ is the wavelength in vacuum at the center frequency.

**Table 5:** Comparison with other antenna temperature sensors.

Refs.	Type	Center Frequency	Temperature Range	R^2^
[43]	DRA	2.95 GHz	20~370 °C	-
[44]	Patch Antenna	2.8 GHz	0~100 °C	0.9984
[45]	Hybrid Antenna	1 GHz	20~50 °C	0.9991
Proposed	DRA	5.25 GHz	−20~50 °C	0.9936

“-” refers to the parameter that does not exist.

## Data Availability

Samples of the compounds are available from the authors.
